# Are there differences in the clinical and laboratory features of patients with seronegative primary Sjögren’s syndrome?

**DOI:** 10.55730/1300-0144.5873

**Published:** 2024-10-07

**Authors:** Müçteba Enes YAYLA, Rahime AKSOY, Emine USLU, Zehra KARAMAN, Recep YILMAZ, Ahmet İLBAY, Nilgün GÖVEÇ GIYNAŞ, Abdulbaki GAYDAN, Ahmet USTA, Yeter MAHMUTOĞLU, Aşkın ATEŞ, Murat TURGAY

**Affiliations:** 1 Department of Rheumatology, Faculty of Medicine, Ankara University, Ankara, Turkiye; 2 Department of Hematology, Faculty of Medicine, Ankara University, Ankara, Turkiye

**Keywords:** SS-A antibody, SS-B antibody, rheumatoid factor, antinuclear antibody, C4 complement

## Abstract

**Background/aim:**

The objective of this study was to demonstrate the commonalities and distinctions between patients with seronegative and seropositive primary Sjögren’s syndrome (pSS).

**Materials and methods:**

The records of 399 patients with pSS seen between January 2010 and June 2023 were retrospectively examined. Patients with negative antiSSA/Ro, antiSSB/La, ANA, and RF antibodies comprised the seronegative group, while patients with at least one positive antibody were included in the seropositive group.

**Results:**

The most common clinical features between the groups were arthralgia (81.2%), arthritis (11.5%), hematological involvement (19.8%), and pulmonary involvement (11.8%). In 41 patients (10.3%), no autoantibody positivity was detected. The number of patients with at least one extraarticular involvement was statistically more frequent in the seropositive group (p = 0.011). Dry mouth was found to be more prevalent among seronegative patients (p = 0.003). While hyperimmune gammaglobulinemia exhibited a higher prevalence within the seropositive group (p = 0.004), the occurrence of reduced complement levels was at similar rates in both groups. All deaths were observed exclusively within the seropositive group (17/358, 4.7%). No difference was observed between the two groups concerning mortality (p = 0.237) and malignancies (seropositive group: 9/358, 2.5% vs. seronegative group: 3/41, 7.3%, p = 0.115). There was a statistically significant association between low C4 levels (OR = 2.99 [1.09–8.16], p = 0.045 in model 1, OR = 3.10 [1.14–8.42], p = 0.022 in model 2), and the extraarticular findings.

**Conclusion:**

While hematological, renal, pulmonary, and neurological involvements are observed with similar frequency in both seronegative and seropositive pSS patients, the presence of extraarticular manifestations was more common in seropositive patients. Additionally, there was a relationship between extraarticular involvement and low C4 levels.

## 1. Introduction

Primary Sjögren’s syndrome (pSS) is a systemic autoimmune disorder distinguished by lymphocytic infiltration of the exocrine glands that predominantly impacts middle-aged women [1]. While dryness of the mouth and eyes are the typical clinical manifestations, it is important to note that extraglandular involvement, including conditions like interstitial lung disease, central nervous system involvement, and autoimmune cytopenia, may also manifest during the course of this illness [2].

Autoantibodies that emerge due to B cell activation constitute a distinctive hallmark of pSS. Immunological markers play a pivotal role in both the diagnosis and classification of the disease [3,4]. The presence of antinuclear antibodies (ANA) can be detected in approximately 80% of pSS patients, antiSSA/Ro in 75%, and antiSSB/La and rheumatoid factor (RF) in 50% [5]. Constitutional symptoms, cutaneous manifestations, renal abnormalities, and hematological findings are more prevalent in patients with a positive status for antiSSA/Ro antibodies. Glandular manifestations and lymphadenopathy may occur concurrently with these indications in patients positive for antiSSB/La antibodies [5]. In line with the possible relationship between the presence of antibodies and the clinical and laboratory findings, the question arose whether seronegative pSS patients may have different characteristics. However, seronegativity in pSS has been defined differently in many studies. While certain studies exclusively focused on the absence of antiSSA/Ro and antiSSB/La antibodies [6,7], others also included the negativity of ANA [8]. There are few studies exploring the concept of quadruple negativity in conjunction with RF [8,9]. This study was designed to examine whether disparities exist in the demographic, clinical, and laboratory characteristics of patients with pSS who exhibit quadruple (ANA, antiSSA/Ro, antiSSB/La, and RF) antibody negativity.

## 2. Materials and methods

Between January 2010 and June 2023, 423 patients with pSS applied to the outpatient clinic of the Rheumatology Department of the Ankara University Faculty of Medicine. Of these patients, 399 whose ANA, RF, and extractable nuclear antigen profiles were assessed were included in the study. The 2016 pSS classification criteria of the American College of Rheumatology (ACR)/European League against Rheumatism (EULAR) was used to classify all patients [4]. All patients were over the age of 18 and had no other autoimmune connective tissue disease.

Patients with negative antiSSA/Ro, antiSSB/La, ANA, and RF antibodies comprised the seronegative group, while patients with at least one antibody positivity were included in the seropositive group. The clinical characteristics of the patients were investigated retrospectively using digital file data. The disease onset age was defined as the time of the first symptom considered attributable to pSS. The follow-up period was determined as the duration between first and last visits. The organ and system involvements associated with pSS were defined using ESSDAI parameters [10]. Extraarticular involvement was determined as the presence of system or organ involvement other than the glandular and musculoskeletal systems. Ocular staining scores ≥ 5 on at least one eye or Schirmer scores ≤ 5 mm/5 min on at least one eye were considered abnormal ocular test results [4,11,12]. Unstimulated whole saliva flow rate ≤ 0.1 mL/min was regarded as an abnormal saliva flow test [13]. RF, immunoglobulin (Ig) G levels, C3, C4, ANA, antiSSA/Ro, and SSB/La values were obtained from the patient files. Indirect immunofluorescence was employed to detect ANA in HEp-2 cells. Sera with an antibody titer of 1:100 or higher were classified as positive. RF was evaluated nephelometrically, and RF above the laboratory upper limit of 14 IU/mL was considered positive.

Permission for the current study was received from the Ankara University Faculty of Medicine Ethics Committee on 25 July 2023 with the decision number 2023/446.

### 2.1. Statistical analysis

SPSS v21 (SPSS, Chicago, USA) was used to analyze the data. Frequencies and percentages are used for categorical data. The quantitative data are presented as medians and interquartile ranges (IQR) (nonnormally distributed) or as means with standard deviation (normally distributed). As appropriate, Fisher’s exact test or a chi-squared test was used to compare categorical data. Either the Mann–Whitney U test or Student’s t test was used to compare the quantitative data. A multivariable analysis on potential factors that had been identified in the univariate analyses was conducted via logistic regression modeling, with the aim of identifying associated factors for extraarticular involvement. The odds ratios (OR) calculated from these analyses were defined using 95% confidence intervals. The Hosmer–Lemeshow test was used to assess goodness-of-fit. Overall, p < 0.05 was regarded as statistically significant.

## 3. Results

A total of 399 patients underwent evaluation. Of these patients, 94.7% were female (378/399), the mean age at time of diagnosis was 51.3 ± 12.3 years, and the median duration of follow-up was 5.2 (IQR 7.4) years. In 41 patients (10.3%), no autoantibody positivity was detected, and these patients were considered the seronegative group. The most common clinical features were arthralgia (81.2%), arthritis (11.5%), hematological involvement (19.8%), and pulmonary involvement (11.8%). There was at least one extraarticular involvement in 37.8% of the patients (Table 1). During follow-up, malignancy occurred in 12 patients (Table 2), and 17 patients died.

### 3.1. Comparison of seronegative and seropositive patients

Regarding clinical characteristics, both groups exhibited comparable features. However, the number of patients with at least one extraarticular involvement was statistically more frequent in the seropositive group (p = 0.011). Dry mouth was found to be more prevalent among seronegative patients (p = 0.003); nevertheless, the presence of abnormal saliva flow did not reach statistical significance (p = 0.068). The symptoms and indications of ocular dryness were similar in both groups. While hyperimmune gammaglobulinemia was more prevalent within the seropositive group (p = 0.004), reduced complement levels occurred at similar rates in both groups (Table 1).

All deaths that occurred during the follow-up period were exclusively within the seropositive group (17/358, 4.7%). No differences were observed between the two groups concerning mortality (p = 0.237) and malignancies (seropositive group: 9/358, 2.5% vs. seronegative group: 3/41, 7.3%, p = 0.115) (Table 2).

Upon evaluating the treatments administered during the follow-up period, it was noted that the use of glucocorticoids was more prevalent within the seropositive group, albeit without reaching statistical significance (p = 0.095). There was no difference between the two groups in terms of other treatments (Table 3).

### 3.2. Factors associated with extraarticular involvement

Univariate and multivariable logistic regression analyses were conducted to assess the potential associations between extraarticular involvement and clinical as well as serological factors (Tables 4 and 5). There was a statistically significant association between the extraarticular findings and low C4 levels (OR = 2.99 [1.09–8.16], p = 0.045 in model 1, OR = 3.10 [1.14–8.42], p = 0.022 in model 2) (Table 5).

## 4. Discussion

In the diagnostic evaluation of pSS, the results of minor salivary gland biopsies and autoantibody tests are important to consider in addition to glandular symptoms. Autoantibodies have consistently been an integral component of the classification criteria for pSS over the past three decades. In the European criteria of 1993, ANA, antiSSA/Ro, antiSSB/La, and RF were all included; however, ANA and RF were later excluded from the classification criteria, followed by the exclusion of antiSSB/La [4,5,14,15]. Despite exclusion from the classification criteria, the relationship between autoantibodies and clinical features has been a frequent subject of research in pSS. In this study, we investigated whether the absence of the four most common autoantibodies in pSS patients, which have been included in the diagnostic or classification criteria for the last 30 years, makes a clinical difference. In the current study, seronegativity was found in 10.9% of patients. Extraarticular involvement and hypergammaglobulinemia were less common and dry mouth was more common in seronegative patients.

After an extensive literature search, two studies were found that assess the impact of ANA, RF, antiSSA/Ro, and antiSSB/La antibodies in pSS [8,9]. Chatsiz et al. found quadruple autoantibody negativity in 4.6% of patients, and when their patients with quadruple autoantibody negative were compared with antiSSA/Ro positive controls, regardless of RF or antiSSB/La status, persistent lymphadenopathy and lymphoma were observed to be less common in the group with quadruple antibody negativity [8]. They also showed that dry eye was more common in patients with quadruple autoantibody negative compared to antiSSA/Ro positive and RF negative patients. In the same study, when triple autoantibody negative (ANA positive, but antiSSA/Ro, SSB/La, and RF negative) patients were compared with quadruple autoantibody negative patients, the frequency of lymphadenopathy and lymphoma was found to be lower in the quadruple autoantibody negative group [8]. In research conducted by Yazısız et al., hypergammaglobulinemia and pilocarpine use were found to be less common in patients with quadruple autoantibody negativity [9]. In that study, organ and system involvements were evaluated separately, as well as extraglandular findings collectively, and no difference was detected between the two groups [9].

In this study, the complaint of dry mouth was observed more frequently in the seronegative group. The decision was made to evaluate extraarticular involvement instead of extraglandular involvement because it is thought to better reflect systemic visceral involvement. Extraarticular involvement was frequently identified in the seropositive group. Insufficient data pertaining to lymphadenopathy prevented evaluation of patients in this regard; however, the number of patients who developed lymphoma was quite low. Also, the retrospective nature of this study and the short median follow-up period of the patients prevented an investigation into the development and frequency of malignancy. Therefore, the effect of seronegativity on lymphoma development could not be explored. However, the data show no difference between the two groups in terms of malignancy. In terms of laboratory characteristics, hypergammaglobulinemia was identified more frequently in the seropositive group. Similar to previous studies, hypocomplementemia was not found to be different in the seronegative group [8,9].

Despite the relatively limited number of studies reporting quadruple antibody negativity, there exist numerous investigations examining the clinical association of concomitant negativity for antiSSA/Ro and antiSSB/La antibodies [6,7,16]. It has been reported that antiSSA/Ro and antiSSB/La negative patients have shorter disease durations and are more likely to be male and have interstitial lung disease. Hematological involvement, hypergammaglobulinemia, and hypocomplementemia were revealed to be less common in these patients [6,7]. Additionally, lymphoma development is observed less frequently in seronegative patients [5,6,17]. A study examining peripheral neuropathy reported that while small fibers were primarily affected in seronegative patients, both axonal and small fibers were affected in seropositive patients [18]. All these studies indicate that seropositive and seronegative patients may show different clinical and laboratory features. In particular, extraglandular involvement and lymphoma risk may be associated with the presence of autoantibodies. In this study, organ and system involvement was similar in the seronegative and seropositive groups. However, the naturally small number of patients in the seronegative group may have hindered accurate observation of the true frequency of some extraglandular involvement findings.

Several clinical and laboratory characteristics have been identified in patients with pSS in relation to extraglandular involvement. Relationships have been identified between renal involvement and early disease onset; neurological involvement and disease duration; between low C4, hypergammaglobulinemia, interstitial lung disease, and cough; and between dyspnea, Raynaud’s phenomenon, antiSSA/Ro52, leukocytopenia, and low C3, hypergammaglobulinemia, and antiSSA/Ro [19–21]. Furthermore, the presence of cryoglobulins appears to be associated with extraglandular manifestations [20]. While cutaneous vasculitis is associated with poor prognosis, severe disease, and systemic involvement, the presence of antiSSA/Ro and/or antiSSB/La antibodies also appears to be related to cutaneous vasculitis [22–24]. In the current study, the multivariable analyses revealed a relationship between extraarticular involvement and only low C4. Additionally, while the univariate analyses identified a statistical relationship between the presence of antiSSA/Ro52 with antiSSB/La and extraarticular involvement, this relationship could not be demonstrated in multivariable analyses despite the result of p = 0.051 (Tables 4 and 5). It is known that B lymphocytes play a significant role in the pathogenesis of pSS. Dysregulation in B lymphocytes has a notable impact on the development of autoimmunity and extraglandular involvement. Interleukin-6 released from dysregulated B lymphocytes is a key factor in the production of autoantibodies [25–27]. It has also been shown that the increase in B cell activating factor in both serum and salivary glands correlates with disease activation and antiSSA/Ro and antiSSB/La levels [28]. Considering this knowledge, seronegative pSS patients might be expected to exhibit a distinct and possibly milder clinical phenotype when compared to pSS patients who are positive for autoantibodies.

The current study has some limitations, such as being retrospective and conducted at a single center. The relatively lower number of seronegative patients compared to the seropositive group, inherently associated with the nature of the disease, also emerges as a significant limitation. Additionally, one drawback of using retrospective data is the limited ability to detect malignancies diagnosed elsewhere that developed during follow-up. This could be a reason for the lower frequency of lymphomas in the patients. Furthermore, our capacity to provide commentary on mortality was also diminished by the relatively brief median follow-up period. Nevertheless, the strength of this study is that it is one of the few studies investigating the negativity of the four most common autoantibodies in pSS patients.

In conclusion, while hematologic, renal, pulmonary, and neurological involvements are observed with similar frequencies in both seronegative and seropositive pSS patients, the presence of any extraarticular manifestations was more common in seropositive individuals. Additionally, there was a relationship between extraarticular involvement and low C4 levels.

## Figures and Tables

**Table 1:** Comparison of seronegative and seropositive pSS patients.

	All patients n = 399	Seronegative group n = 41	Seropositive group n = 358	p
Sex, female, n(%)	378 (94.7)	38 (92.7)	340 (95.2)	0.465
Age at diagnosis, year†	51.3 ± 12.3	53.2 ± 10.5	51 ± 12.7	0.218
Follow-up time, year¥	5.2 [7.4]	6.3 [5.8]	5 [7.6]	0.519
**Clinical features, n (%)**				
Arthralgia	324 (81.2)	31 (75.6)	293 (81.8)	0.333
Arthritis	46 (11.5)	5 (12.2)	41 (11.5)	0.800
Extraarticular involvement	151 (37.8)	8 (19.5)	143 (39.9)	0.011
Hematological involvement	79 (19.8)	5 (12.2)	74 (20.7)	0.197
Pulmonary involvement	47 (11.8)	3 (7.3)	44 (12.3)	0.450
Renal involvement	13 (3.3)	0	13 (3.6)	0.378
Skin involvement	20 (5)	1 (2.4)	19 (5.3)	0.708
Peripheral nervous system involvement	12 (3)	0	12 (3.4)	0.621
Central nervous system involvement	12 (3)	0	12 (3.4)	0.621
Myositis	3 (0.7)	0	3 (0.8)	>0.999
**Glandular features**				
Dry mouth, n (%)	329/394 (83.5)	40/40 (100)	289/354 (81.6)	0.003
Dry eyes, n (%)	307/380 (80.8)	35/39 (89.7)	272/341 (79.8)	0.134
Abnormal salivary flow test, n (%)	185/259 (71.4)	28/33 (84.8)	157/226 (69.5)	0.068
Abnormal ocular test, n (%)	151/191 (79.1)	21/24 (87.5)	130/167 (77.8)	0.277
Focus score on minor salivary gland biopsy ¥	1 [1], min:0, max:6	1 [1] min:1, max:3	1 [2] min:0, max:6	0.133
**Serological features, n (%)**				
ANA positivity	310/397 (78.1)	0	310/356 (87.1)	-
RF presence	97/388 (25)	0	97/347 (28)	-
Anti SSA (Ro52 or 60) presence	288/399 (72.2)	0	288/358 (80.4)	-
Anti SSA/Ro52 presence	209/399 (52.4)	0	209/258 (58.4)	-
Anti SSA/Ro60 presence	255/399 (63.9)	0	255/358 (71.2)	-
Anti SSB presence	99/399 (24.8)	0	99/358 (27.7)	-
Hypergammaglobulinemia	72/230 (31.3)	1/22 (4.5)	71/208 (34.1)	0.004
Low C3	47/283 (16.6)	4/27 (14.8)	43/256 (16.8)	>0.999†
Low C4	33/283 (11.7)	2/28 (7.1)	31/255 (12.2)	0.755†

ANA = antinuclear antibody, n = number of patients, RF = rheumatoid factor.

† Data are presented as mean and standard deviation.

¥ Data are presented as median and interquartile range.

**Table 2:** Malignancy characteristics of pSS patients.

		Seronegative group n = 41	Seropositive group n = 358
**Malignancies**		3 (7.3)	9 (2.5)
	Basal cell carcinoma	0	2 (0.6)
	Breast cancer	1 (2.4)	2 (0.6)
	Lymphoma	1 (2.4)	0
	Mycosis fungoides	1 (2.4)	0
	Papillary thyroid carcinoma	0	2 (0.6)
	Squamous cell carcinoma of the skin	0	1 (0.3)
	Colon carcinoma	0	1 (0.3)
	Myelofibrosis	0	1 (0.3)

Data are presented as number and percentile, n = number of patients.

**Table 3:** Treatment features of pSS patients.

	All patients n = 399	Seronegative Group n = 41	Seropositive Group n = 358	p
Hydroxychloroquine	358 (89.9)	39 (95.1)	319 (89.4)	0.407
Glucocorticoids	101 (25.4)	6 (14.6)	95 (26.6)	0.095
Azathioprine	34 (9.5)	3 (7.3)	34 (9.5)	>0.999
Pilocarpine	32 (8)	4 (9.8)	28 (7.8)	0.557
Methotrexate	27 (6.8)	3 (7.3)	24 (6.7)	0.750
Mycophenolate mofetil	16 (4)	2 (4.9)	14 (3.9)	0.675
Cyclophosphamide	12 (3)	1 (2.4)	11 (3.1)	>0.999
Rituximab	9 (2.3)	0	9 (2.5)	0.607
Cyclosporin	2 (0.5)	0	2 (0.6)	>0.999

n = number of patients.

**Table 4:** Factors associated with extraarticular involvement in the univariate analysis.

	Univariate analysis OR [CI]	p
Sex, male	1.87 [0.77–4.51]	0.164
Age at diagnosis, year	0.99 [0.98–1.01]	0.439
ANA positivity	1.30 [0.79–2.14]	0.307
RF presence	1.21 [0.75–1.93]	0.432
Anti SSA/Ro presence	2.08 [1.28–3.37]	0.003
Anti SSA/Ro52 presence	2.28 [1.50–3.46]	<0.001
Anti SSA/Ro60 presence	1.73 [1.18–2.67]	0.014
Anti SSA/Ro (without SSB) presence	1.99 [1.23–3.22]	0.005
Anti SSB/La presence	2.51 [1.58–3.99]	<0.001
Anti SSB/La and SSA/Ro presence	2.57 [1.60–4.14]	<0.001
Anti SSB/La (without SSA) presence	1.24 [0.27–5.60]	0.783
Hypergammaglobulinemia	1.73 [0.98–3.04]	0.058
Low C3	1.87 [0.97–3.46]	0.060
Low C4	3.23 [1.47–7.01]	0.003

ANA = antinuclear antibody, CI = confidence interval, OR = odds ratio.

**Table 5:** Factors associated with extraarticular involvement in the multivariable analysis.

	Multivariable analysis Model 1 OR [CI]*	p	Multivariable analysis Model 2 OR [CI]**	p
Sex, male	1.81 [0.38–3.29]	0.846	1.14 [0.39–3.35]	0.811
Anti SSA/Ro52 presence	1.39 [0.75–2.57]	0.291	-	-
Anti SSB/La presence	1.84 [0.92–3.67]	0.086	-	-
Anti SSA/Ro52 and SSB/La presence	-	-	1.98 [0.99–3.94]	0.051
Hypergammaglobulinemia	1.20 [0.63–2.29]	0.574	1.23 [0.65–2.35]	0.522
Low C3	1.08 [0.48–2.43]	0.846	1.06 [0.47–2.36]	0.893
Low C4	2.99 [1.09–8.16]	0.045	3.10 [1.14–8.42]	0.022

CI = confidence interval, OR = odds ratio.

* chi-squared 5.296, p = 0.506 for model 1

** chi-squared 5.906, p = 0.563 for model 2
